# Neoadjuvant tislelizumab combined with chemotherapy in locally advanced oral or oropharyngeal squamous cell carcinoma: a real−world retrospective study

**DOI:** 10.3389/fimmu.2023.1282629

**Published:** 2023-11-14

**Authors:** Wen-Jie Wu, Qian Liu, Pu-Gen An, Lin Wang, Jian-Yun Zhang, Yan Chen, Tong Zhang, Jie Zhang

**Affiliations:** ^1^ Department of Oral and Maxillofacial Surgery, Peking University School and Hospital of Stomatology, Beijing, China; ^2^ National Center of Stomatology & National Clinical Research Center for Oral Diseases, Beijing, China; ^3^ Central Laboratory, Peking University School and Hospital of Stomatology, Beijing, China; ^4^ Department of Oral Pathology, Peking University School and Hospital of Stomatology, Beijing, China; ^5^ Departmet of Oncology, Xiyuan Hospital of China Academy of Chinese Medical Sciences, Beijing, China

**Keywords:** neoadjuvant, immunotherapy, chemotherapy, head and neck, squamous cell carcinoma

## Abstract

**Objectives:**

The treatment of locally advanced oral or oropharyngeal squamous cell carcinoma (LAOOPSCC) is surgery and radiotherapy or chemoradiotherapy but with unsatisfactory survival rate. Neoadjuvant programmed death-1 (PD-1) therapy are being used in several clinical trials. Therefore, in this retrospective study we aimed to determine the feasibility of neoadjuvant tislelizumab plus chemotherapy followed by surgery for LAOOPSCC.

**Materials and methods:**

The clinical data of 33 patients with LAOOPSCC who received neoadjuvant PD-1 inhibitors and chemotherapy between April 2021 and October 2022 were retrospectively analyzed. Patients with stage III-IV LAOOPSCC received tislelizumab, albumin-bound paclitaxel, and cisplatin every 3 weeks (Q3W) for two cycles, followed by surgery and adjuvant radiotherapy or concurrent chemoradiotherapy. A median follow-up period was 20 months.

**Results:**

The objective response rate (ORR) was 66.7%, with the major pathological response (MPR) rate at 54.5%, and the pathological complete response (pCR) rate was 33.3%. Sixteen patients underwent limited surgeries, and 15 patients were remitted from undergoing mandibulectomy and 9 patients were remitted from undergoing near total glossectomy or total glossectomy. A significant difference in the overall survival (OS) and disease-free survival (DFS) was observed in patients who achieved major pathological response (MPR) than who did not. The most common adverse events in neoadjuvant therapy were alopecia, decreased appetite or anorexia, leukopenia, and fatigue.

**Conclusion:**

Neoadjuvant PD-1 inhibitors combined with chemotherapy are feasible and safe, with a high pathological response and possible organ preservation in oral or oropharyngeal squamous cell carcinoma. However, further studies with a larger cohort of patients and longer follow-up period is required to strengthen our findings and evaluate the survival benefits of the treatment.

## Introduction

Oral and oropharyngeal squamous cell carcinomas account for more than 65% of head and neck squamous cell carcinomas (HNSCC) (1). The mainstay treatment of locally advanced HNSCC is surgery and adjuvant radiotherapy or chemoradiotherapy (2), with the 5-year overall survival rate of 40 – 50% (3, 4). Studies have attempted to improve this relatively low survival rate using neoadjuvant therapy. In a randomized phase III trial, docetaxel, cisplatin, and fluorouracil (TPF) chemotherapy which includes three chemotherapy agents, docetaxel, cisplatin, and fluorouracil, was used as neoadjuvant therapy for locally advanced oral squamous cell carcinoma before surgery, however the results did not demonstrate improvement in survival compared with standard treatment (5).

Programmed death-1 (PD-1) inhibitors are being used as first- and second-line treatments for recurrent or metastatic HNSCC (6, 7). The objective response in the pembrolizumab plus chemotherapy group (36%) was observed to be higher than that in the pembrolizumab alone group (17%) amongst the total population (7). In addition, the feasibility of neoadjuvant immunotherapy plus chemotherapy has been previously evaluated in non–small cell lung cancer (8, 9). Several trials on neoadjuvant anti–PD-1/PD-1 ligand 1 (PD-L1) administered to locoregionally advanced, resectable HNSCC have provided promising initial results (10). Moreover, compared to trials of neoadjuvant PD-1 inhibitor monotherapy, fewer trials of PD-1 inhibitors plus chemotherapy exist (10).

Tislelizumab is an anti- PD-1 monoclonal immunoglobulin G 4 antibody, approved for usage in the treatment of nine cancer types in multiple clinical trials (11). This retrospective study aimed to determine the feasibility of neoadjuvant tislelizumab plus chemotherapy, followed by surgery in locally advanced oral or oropharyngeal squamous cell carcinoma.

## Materials and methods

### Study design, patients and treatment

Patients were enrolled in this retrospective study from April 2021 to October 2022 according to the following inclusion criteria: (a) patients with stage III or IV oral or oropharyngeal squamous cell carcinoma, (b) patients who received neoadjuvant tislelizumab plus chemotherapy followed by surgery, and (c) had an Eastern Cooperative Oncology Group (ECOG) performance status of 0–1. The exclusion criteria were as follows: (a) previous tumors, (b) preoperative radiotherapy, (c) distant metastasis, and (d) ineligibility for PD-1 inhibitors plus chemotherapy. A total of 33 consecutive patients were included in the study. The basic patient information is shown in **Table 1**.

**Table 1:** Baseline characteristics.

		*N (%)*
**Age**	Median	59 (range 38,75)
**Sex**	Male	27 (81.8%)
	Female	6 (18.2%)
**ECOG PS**	0	20 (60.6%)
	1	13 (39.4%)
**Tumor sites**	Oral Cavity	18 (54.5%)
	Oropharynx	15 (45.5%)
**T**	T1	2 (6.1%)
	T3	5 (15.1%)
	T4a	23 (69.7%)
	T4b	3 (9.1%)
**N**	N0	14 (42.4%)
	N1	5 (15.2%)
	N2	4 (12.1%)
	N3b	10 (30.3%)
**M**	M0	33 (100%)
	M1	0
**AJCC stage (the eighth edition)**	III*	7 (21.2%)
	IVA	15 (45.5%)
	IVB	11 (33.3%)
**ENE**	Positive	6 (18.2%)
	Negative	27 (81.8%)
**Smoking**	No	11 (33.3%)
	Yes	22 (66.7%)
**Drinking**	No	13 (39.4%)
	Yes	20 (60.6%)
**p16 status**	Positive	3 (9.1%)
	Negative	30 (90.9%)
**PD-L1 CPS**	<1	4 (12.1%)
	20>CPS≥1	19 (57.6%)
	≥20	10 (30.3%)
**PD-L1 TPS**	<1%	8 (24.2%)
	≥1%	25 (75.8%)
**Imaging evaluation**	CR	4 (12.1%)
	PR	18 (54.6%)
	SD	11 (33.3%)
**Pathologic assessment of resected specimens**	Non-MPR	15 (45.5%)
	MPR	18 (54.5%)
**State**	Alive	29 (87.9%)
	Death	4 (12.1%)

ECOG PS: ECOG, Eastern Cooperative Oncology Group performance status score;

*Three patients with locally advanced HPV-associated oropharyngeal carcinoma were classified as stage III rather than stage IV according to the American Joint Committee on Cancer (AJCC) Cancer Staging Manual, eighth edition.

Patients were treated with two cycles of tislelizumab 200 mg, albumin-bound paclitaxel 260 mg/m^2^, and cisplatin 75 mg/m^2^ with a three-week interval between each cycle, during the neoadjuvant therapy. Computed tomography (CT) and magnetic resonance imaging (MRI) of the head and neck were performed before treatment and two–three weeks after completion of neoadjuvant therapy for efficacy evaluation. Surgery was performed after efficacy evaluation by a multidisciplinary team (MDT) and patients were recommended to be treated with adjuvant radiotherapy. Patients with positive margins or extranodal extension (ENE) were recommended to undergo adjuvant radiotherapy with a regimen of cisplatin 40 mg/m^2^ weekly.

### PD-L1 immunohistochemistry

Specimens for PD-L1 immunohistochemistry were obtained via biopsy of the primary tumor before treatment. The status of PD-L1 expression was measured using the PD-L1 IHC 22C3 pharmDx assay kit (Agilent Technologies, Santa Clara, CA, USA) by following manufacturer’s instructions.

### Outcome definition

Responses were assessed based on the Response Evaluation Criteria in Solid Tumors (RECIST1.1). Objective response rate (ORR) is the sum of the proportions of complete response (CR) and partial responses (PR). Major pathological response (MPR) was defined as ≤10% residual viable tumor cells in the primary tumor. A pathological complete response (pCR) was defined by the absence of viable tumor cells in the primary tumor and sampled lymph nodes. The pathological responses were independently judged by two experienced pathologists. Disease-free survival (DFS) was defined as the period from neoadjuvant therapy to the latest follow-up, recurrence, distant metastasis, or death from any cause. Overall survival (OS) was defined as the period from neoadjuvant therapy to the latest follow-up, or death from any cause during the follow-up.

### Statistical analysis

The Kaplan–Meier method was used to analyze of DFS and OS. The categorical variables were compared using Chi-square. Between-group differences in survival were determined using the log-rank test. All the analyses were performed using GraphPad Prism (version 9) and IBM SPSS (version 24).

## Results

### Efficacy of neoadjuvant therapy

**Figure 1:**
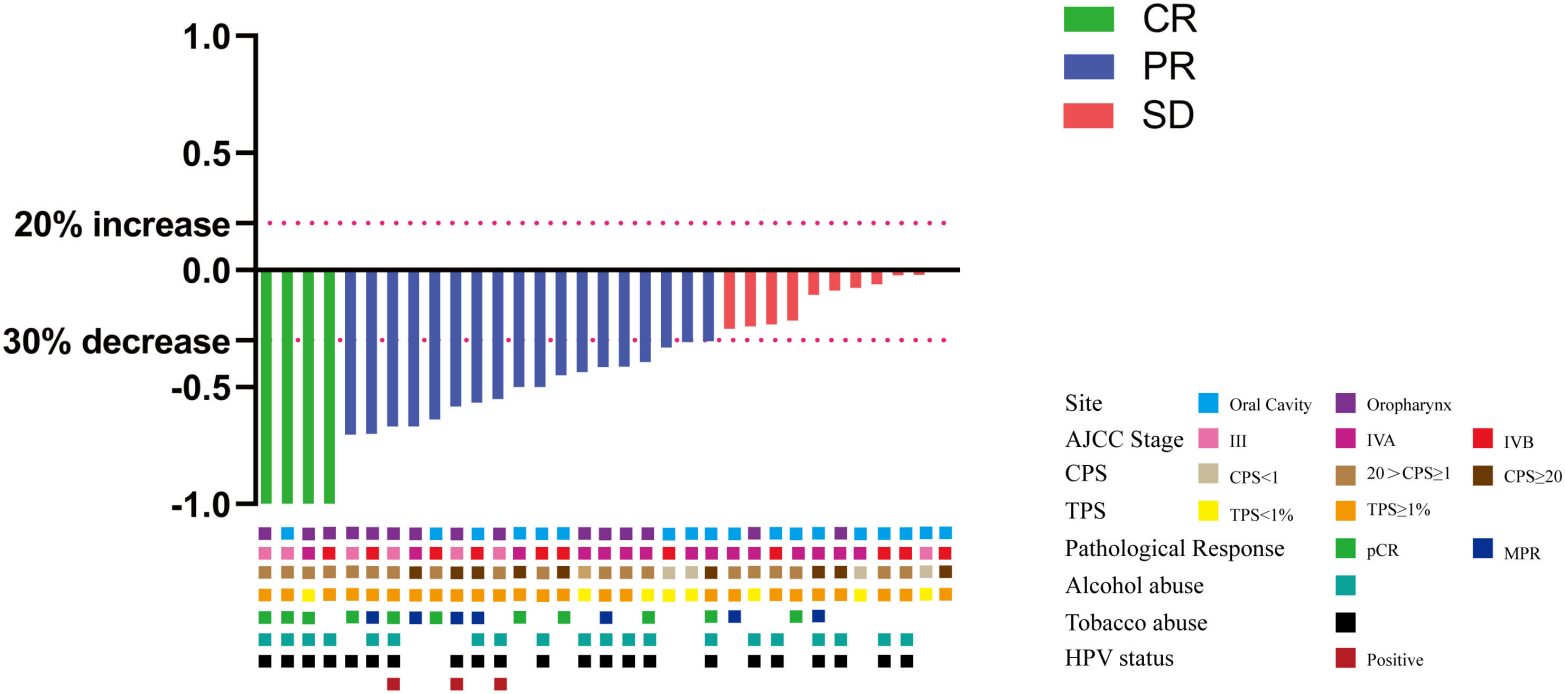
Tumor response in the 33 patients.

Twenty-seven (81.8%) patients complained of symptoms including pain and dysphagia, but all of them reached remission after neoadjuvant therapy. The ORR was 66.7%, achieving CR in 4 patients, PR in 18 patients, stable disease (SD) in 11 patients, and no patients had progressive disease (PD) (**Figure 1**). Eighteen patients had radiographic downstaging in the T stage and seven patients had N stage downstaging. The relationship between categorical variables and ORR/N-ORR is shown in **Table S1**.

**Figure 2:**
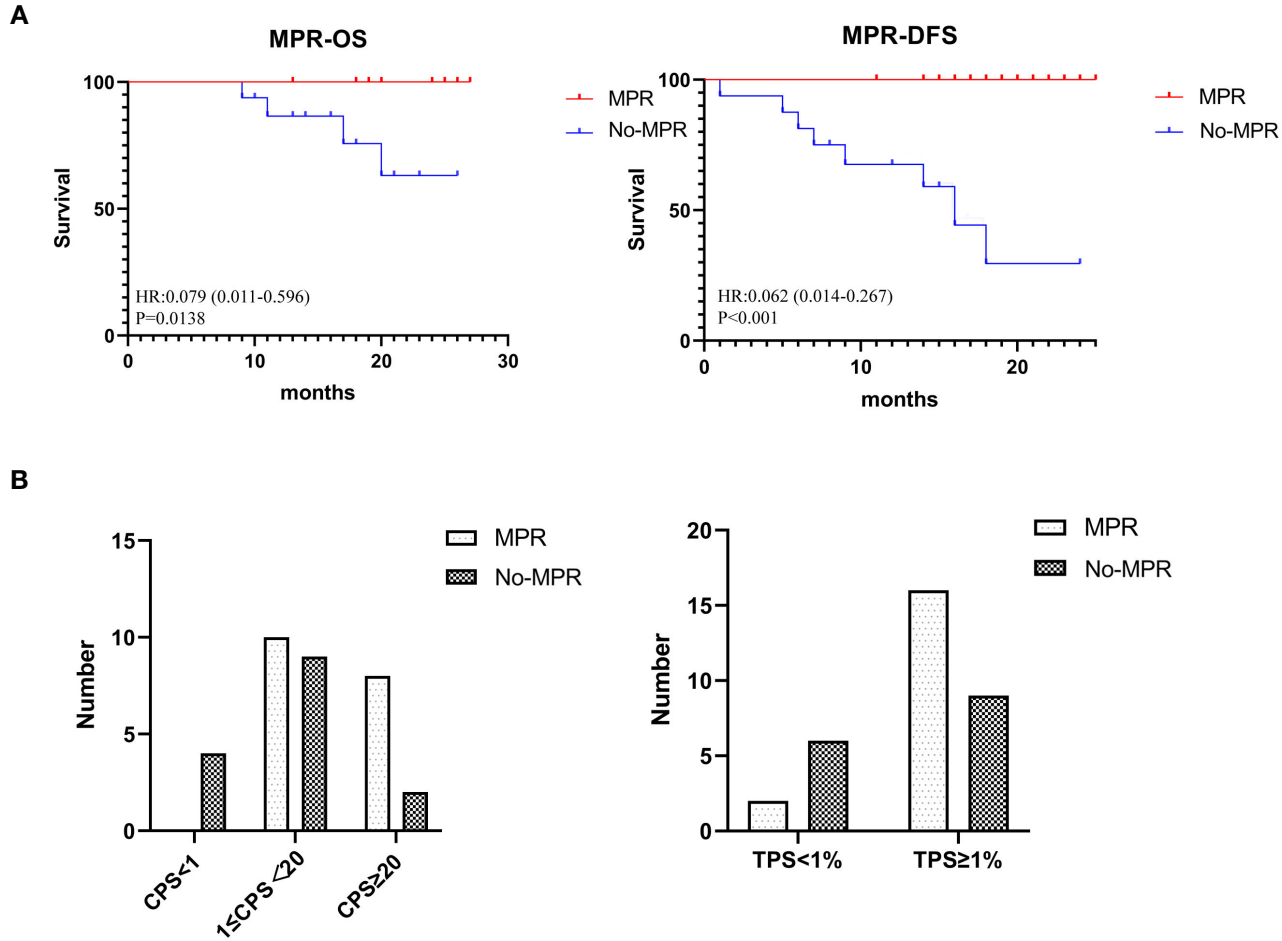
**(A)** Kaplan-Meier analysis showing OS and DFS of MPR. **(B)** The relationship between CPS and TPS and MPR.

The MPR rate was 54.5% (18/33), including 11 patients (33.3%) who achieved pCR.Currently, all patients who achieved MPR are alive. A significant difference was observed in the OS between the MPR and non-MPR groups (*p* =0.0138). In addition, the DFS showed a significant difference between the MPR and non-MPR groups (*p*<.001) (**Figure 2A**). Additionally, differences were observed in MPR between the combined positive score (CPS) and tumor proportion score (TPS) groups (**Figure 2B**). The number of patients achieving MPR were significantly higher when CPS ≥ 1 and TPS ≥ 1% (**Figure 2B**). The pathological results showed that one patient had a positive margin and six patients had ENE, of which four patients had tumor recurrence or metastasis. The relationship between categorical variables and MPR/N-MPR is shown in **Table S2**. The kappa value between ORR and MPR was 0.143, and the p value was 0.026.

### Adverse events of neoadjuvant therapy

We evaluated the safety of neoadjuvant therapy in all the 33 patients and the results are shown in **Table 2**. Grade 3-4 AEs occurred in two patients, which includes grade 3 (G3) leukopenia (3%) and febrile neutropenia (3%) in one patient, G3 hyperglycemia (3%) in another patient. The Grade 1-2 AEs that were observed in patients were alopecia (100%), decreased appetite or anorexia (45.5%), leukopenia (36.4%), fatigue (36.4%) (>25% showed incidence of AEs) and mostly chemotherapy related side effects. None of the patients experienced surgical delays or severe surgical complication.

**Table 2:** Adverse events.

	Grade 1	Grade 2	Grade 3
**Alopecia**	33		
**Decreased appetite or anorexia**	15		
**Leukopenia**	9	3	1
**Fatigue**	11	1	
**Constipation**	8		
**Anemia**	5	2	
**Rash**	2	2	
**Vomiting**	4		
**Hyperglycemia**	4		1
**Increased aminotransferases**	3		
**Increased creatinine**	3		
**Myalgia**	3		
**Febrile neutropenia**			1
**Arthralgia**	1		
**Hypothyroidism**		1	

### Surgery

**Figure 3:**
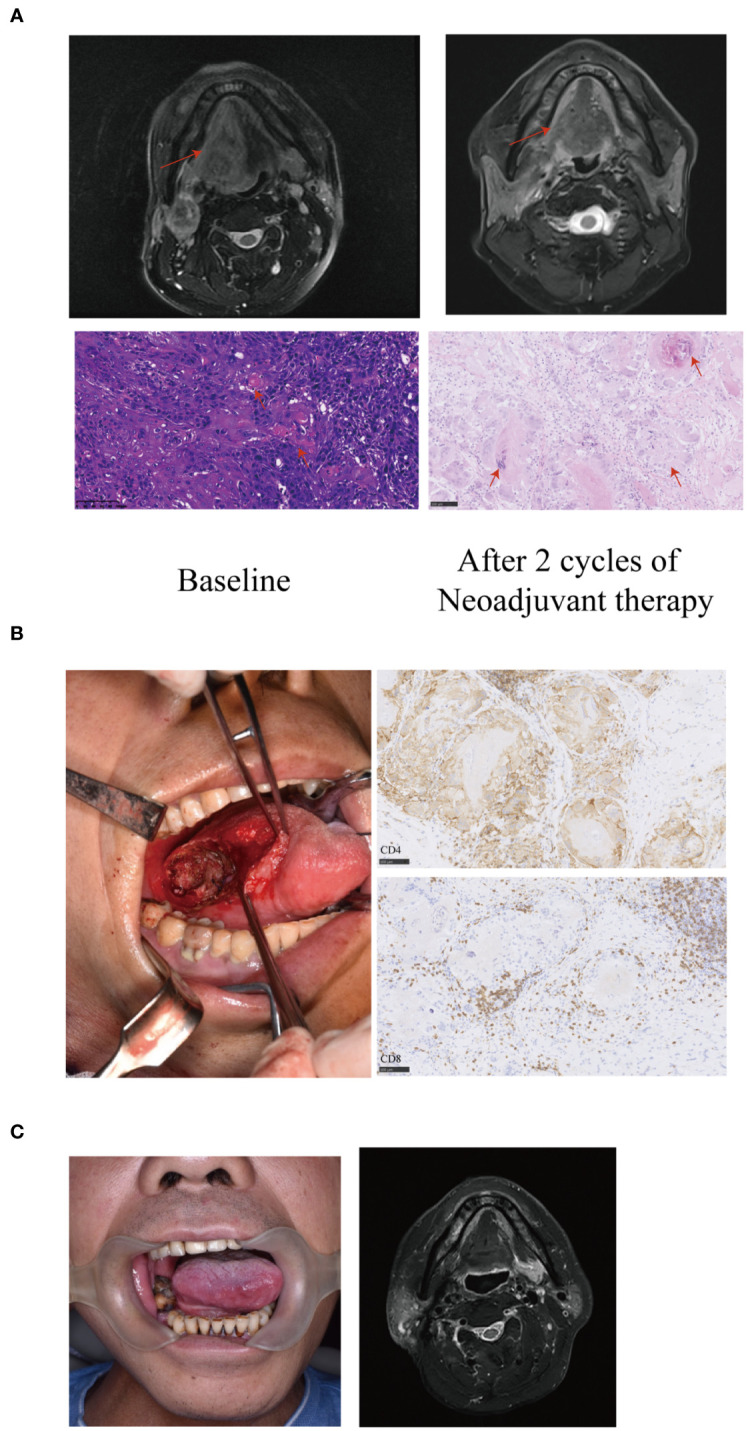
A representative image of a T4aN3bM0 tongue squamous cell carcinoma patient showing significant response to neoadjuvant therapy. **(A)** Baseline magnetic resonance imaging (MRI) showing the tumor extended beyond half of the tongue (arrows) with an extranodal extension (ENE) lymph node of level II (arrows). After two cycles of neoadjuvant therapy, the MRI showed the primary tumor and the lymph node achieved partial response (PR) (arrows). The histopathological examination (20X) of biopsy showed large number of infiltrating tumor nests (arrows). Postoperative hematoxylin and eosin (HE) staining (20X) revealed the disappearance of tumor nests, necrosis and calcification of tumor cells, residual keratin and infiltration of numerous foreign giant cells (arrows). The residual viable tumor cells in the primary tumor were less than 5%. **(B)** The patient underwent limited surgery of the tongue to avoid tracheotomy, free flap reconstruction or facial incisions. Large number of CD4 and CD8 positive cells infiltrating around the tumor nest (immunohistochemistry stain; 20 X) were observed. **(C)** The patient had a satisfactory functional outcome with no sign of recurrence or metastasis with a 27-month follow-up.

All 33 patients underwent an ipsilateral or bilateral neck dissection. Sixteen patients underwent limited surgery for the primary tumor to avoid tracheotomy, free-flap reconstruction, or facial incisions other than neck incisions for neck dissection (**Figure 3**), out of which 12 patients achieved MPR. Moreover, out of the 16 patients, 15 were remitted from undergoing mandibulectomy and 9 were remitted from undergoing near-total glossectomy or total glossectomy. Of 16 patients underwent limited surgery of the primary tumor, one patient who was supposed to undergo total glossectomy underwent limited surgery and experienced recurrence 6 months after surgery. Seven patients relapsed out of the 17 patients who underwent radical surgery, including two who did not undergo adjuvant radiotherapy. Two patients developed bilateral ENE adherence to the internal jugular vein, however after neoadjuvant therapy, the tumors became resectable and both internal jugular veins were retained.

### Adjuvant therapy

Of all the 33 patients, 24 patients were administered adjuvant radiotherapy, 7 patients with high-risk features (positive margins or ENE) were administered adjuvant chemoradiotherapy with a regimen of cisplatin dosed at 40 mg/m^2^ weekly, and 2 patients did not undergo any adjuvant therapy. The 31 patients underwent intensity-modulated radiotherapy (IMRT) using 6 megavolt (mV) photons, scheduled once daily for five days weekly. The clinical target volumes (CTVs) were typically delineated. The GTVtb (Gross tumor volume) was defined as the tumor bed including pretreatment tumor invasion area. The CTV1 was defined as high-risk area encompassing CTVtb plus a 10-20 mm isotropic margin and the levels with pretreatment involved lymph nodes. The CTV2 was defined as low-risk area with suspected subclinical spread. The dose to CTV1 was 60-66 Gy/30 fractions, while CTV2 was administered 54-60 Gy/30 fractions.

### Survival

**Figure 4:**
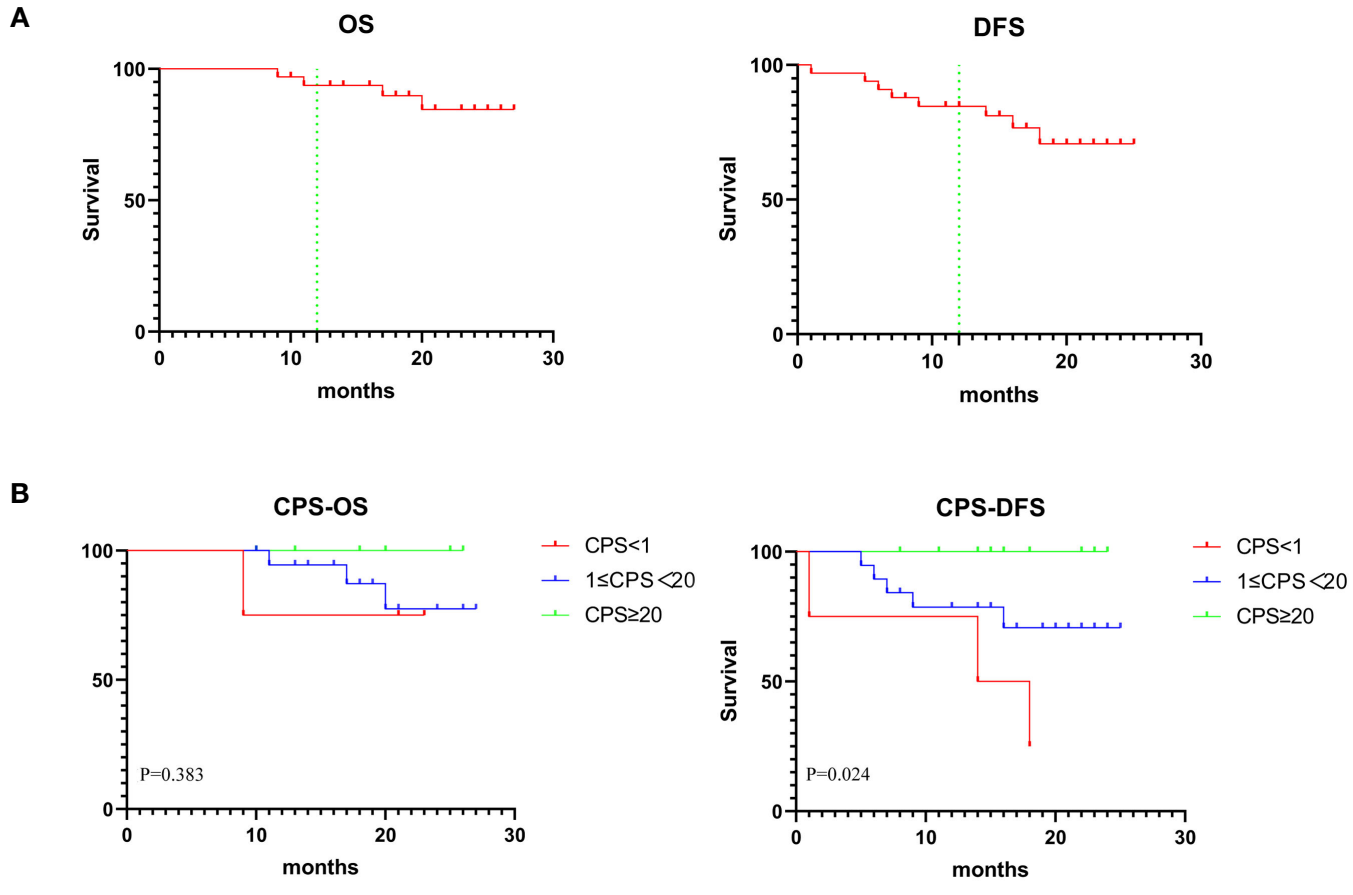
**(A)** Graph showing 1-year OS and DFS in 33 patients. **(B)** Kaplan-Meier analysis showing OS and DFS based on CPS status.

The median follow-up period was 20 months (range, 9–27 months) with the data cutoff in July 2023. **Figure 4A** showed the OS and DFS. The overall one-year survival rate was 93.7% and the one-year disease-free survival rate was 84.6%. Prognostic evaluation based on the PD-L1 CPS status revealed that there was no significant difference in the OS among patients with PD-L1 CPS < 1, 1≤PD-L1 CPS <20, and PD-L1 CPS≥20 (*p*=.3825). But there was a significant difference in the DFS among the three groups (*p*=.0244) (**Figure 4B**). The lack of significant difference in OS across PD-L1 CPS groups could be attributed to the minimal mortality observed, with only one patient decease.

## Discussion

Neoadjuvant immunotherapy has been used in patients with locally advanced HNSCC in several clinical trials. Most of these were single-arm clinical trials providing insufficient evidence and lack of long-term survival data. In addition, the studies have shown that pathological responses in non-small cell lung cancers were associated with long-term survival (8, 9, 12, 13). Accordingly, more attention is being paid to the pathological responses than long-term survival. Single-agent PD-1 inhibitors used as neoadjuvant therapies for locally advanced HNSCC have resulted in low MPR and pCR rates (10). PD-1 plus other checkpoint inhibitors have shown to improve the MPR and pCR rates (10). In IMCISION trial, nivolumab plus ipilimumab showed MPR rate of 35% (14). Moreover, chemotherapy is more likely to shrink tumors in a short time and improve pathological response rates when treated in combination with neoadjuvant therapy. Neoadjuvant PD-1 inhibitors plus chemotherapy have achieved positive results in lung cancers, with higher pathological responses and survival rates (8, 9). Thus, similar to our findings, various previous studies on HNSCC have shown improvement in pathological responses. Zinner et al. evaluated nivolumab, weekly carboplatin, and paclitaxel as neoadjuvant therapies in 32 patients with HNSCC and rates of pCR in HPV-negative and HPV-associated patients were 42% and 50%, respectively with 37% of the patients showing Grade 3 toxicity (15). A single cycle of durvalumab, tremelimumab, cisplatin, and docetaxel as neoadjuvant therapy in 56 patients with stage III-IV HNSCC resulted in a 47% pCR rate with 68% of patients showing Grade 3-4 AE incidence (16). In a phase II trial, 27 patients were treated with neoadjuvant PD-1 inhibitors plus chemotherapy for three cycles followed by surgery, which achieved a 74.1% MPR rate and a 37% pCR rate (17). In another prospective trial, 20 patients with locally advanced oral squamous cell carcinoma were treated with neoadjuvant PD-1 inhibitors plus chemotherapy for two cycles, followed by surgery, which achieved a 60% MPR rate and a 30% pCR rate (18). Similarly, in this study, two cycles of neoadjuvant PD-1 inhibitors plus chemotherapy can result in a satisfactory MPR rate with low toxicity for patients with III-IV oral or oropharyngeal squamous cell carcinoma (78.8%, stage IV). Moreover, PD-L1 immunohistochemistry can predict the pathological responses to PD-1 inhibitors and neoadjuvant chemotherapy which is similar to the conclusions obtained from KEYNOTE-048 and CheckMate-141 clinical trials in recurrent and metastatic HNSCC (6, 7).

In addition to potential survival benefits, neoadjuvant therapy may also result in organ preservation and functional outcomes through limited surgery of the primary tumor or remission surgery, especially for HPV-associated oropharyngeal carcinoma (19). Patients with locally advanced HPV-associated HNSCC have shown an improved response to radiotherapy and survival compared with those with HPV-negative HNSCC (20). Regardless of p16 status, smoking is associated with a worse prognosis in patients with oropharyngeal squamous cell carcinoma (21). In this study, most patients had a history of tobacco and alcohol abuse rather than HPV-associated HNSCC, which may have benefitted from neoadjuvant chemotherapy and PD-1 inhibitors followed by surgery. Additionally, induction chemotherapy followed by radiotherapy for laryngeal carcinoma was performed to avoid laryngectomy (22, 23). In the field of oral squamous cell carcinoma, the mainstay of treatment is surgery and adjuvant radiotherapy or chemoradiotherapy. Advancements in free flap and reconstruction techniques, organ preservation is becoming less important in the field of oral squamous cell carcinoma. A phase II trial enrolled 68 patients who were randomly assigned (1:1) to either surgery followed by adjuvant therapy or two cycles of neoadjuvant therapy (docetaxel, cisplatin, and fluorouracil). The results showed that the DFS and OS were similar, and 47% patients achieved mandibular preservation (24). Patients with radiographic findings of mandibular destruction are usually recommended for mandiblectomy rather than mandibular preservation, based on the MDT discussion however in this study 15 patients were remitted from undergoing mandibulectomy.

Tongue squamous cell carcinoma (SCC) is the most common type of oral cancer. Despite radical surgery for near-total or total glossectomy, the prognosis is poor, with 5-year survival ranging from 25% to 35% for locally advanced tongue cancer (25–28). Most patients undergo free-flap reconstruction and tracheotomy in addition to glossectomy (26, 27). The cosmetic and functional outcomes after glossectomy are vital for surgeons and patients. However, patients with locally advanced tongue cancer have very poor quality of life after near-total or total glossectomy, even if the defect is reconstructed with a free flap (29, 30). Functional morbidities, such as speech and swallowing impairments, affect these patients postoperatively for a long time. Thus, a number of patients refuse to undergo radical surgery for near-total or total glossectomy due to poor prognosis and quality of life in the real world studies. Neoadjuvant therapy offers the possibility of organ preservation as observed in this study where 16 patients underwent limited surgery of the primary tumor, of which 9 patients were remitted from undergoing near total glossectomy or total glossectomy with recurrence only in one patient. Preliminary results in this study suggesting that the pathological response, rather than surgical modality, was the main factor affecting prognosis. Based on the previous results of this study, two prospective studies have been designed and are being conducted (ChiCTR2200056354 and NCT06009861).

Except in case of HPV-associated oropharyngeal carcinoma, limited surgery of the primary tumor was controversial in oral or HPV-negative oropharyngeal squamous cell carcinoma after neoadjuvant PD-1 inhibitors plus chemotherapy. Local control and survival rates were affected by the pathological response status, tumor regression pattern and adjuvant therapy modalities. Although neoadjuvant PD-1 inhibitors plus chemotherapy resulted in high pathological response, tumor regression pattern was not clear in HNSCC. In esophageal squamous cell carcinoma, the pattern of regression toward the lumen (residual tumors mainly in the mucosa and submucosa) is significantly more common after receiving neoadjuvant immunochemotherapy (31). In addition, although the surgery had been downgraded, adjuvant radiotherapy is still necessary, especially in patients with high-risk features (32).

## Conclusions

Neoadjuvant PD-1 inhibitors therapy combined with chemotherapy is feasible and safe, with a high pathological response in oral or oropharyngeal squamous cell carcinoma. Moreover, organ preservation is possible. However, further evidence and longer follow-up periods are required to confirm whether there are any survival benefits.

## Data availability statement

The raw data supporting the conclusions of this article will be made available by the authors, without undue reservation.

## Ethics statement

The studies involving humans were approved by Ethics Committee of Peking University School and Hospital of Stomatology (IRB number: PKUSSIRB-202053013). The studies were conducted in accordance with the local legislation and institutional requirements. Written informed consent for participation in this study was provided by the participants’ legal guardians/next of kin.

## Author contributions

WW: Conceptualization, Funding acquisition, Methodology, Project administration, Software, Supervision, Validation, Visualization, Writing – original draft. QL: Writing – original draft. PA: Data curation, Methodology, Project administration, Writing – original draft. LW: Formal Analysis, Project administration, Validation, Writing – original draft. JYZ: Writing – original draft. YC: Writing – original draft. TZ: Methodology, Software, Validation, Writing – original draft. JZ: Conceptualization, Funding acquisition, Writing – review & editing.
