# Corrigendum: Neoadjuvant tislelizumab combined with chemotherapy in locally advanced oral or oropharyngeal squamous cell carcinoma: a real−world retrospective study

**DOI:** 10.3389/fimmu.2023.1346845

**Published:** 2024-01-05

**Authors:** Wen-Jie Wu, Qian Liu, Pu-Gen An, Lin Wang, Jian-Yun Zhang, Yan Chen, Tong Zhang, Jie Zhang

**Affiliations:** ^1^ Department of Oral and Maxillofacial Surgery, Peking University School and Hospital of Stomatology, Beijing, China; ^2^ National Center of Stomatology & National Clinical Research Center for Oral Diseases, Beijing, China; ^3^ Central Laboratory, Peking University School and Hospital of Stomatology, Beijing, China; ^4^ Department of Oral Pathology, Peking University School and Hospital of Stomatology, Beijing, China; ^5^ Departmet of Oncology, Xiyuan Hospital of China Academy of Chinese Medical Sciences, Beijing, China

**Keywords:** neoadjuvant, immunotherapy, chemotherapy, head and neck, squamous cell carcinoma

**Text Correction**

In the published article, there was an error. Some of the statements in the article about the results in Figure 2A were inappropriate.

A correction has been made to **Results**, *Efficacy of neoadjuvant therapy*, Line 3-9, Paragraph 2, Page 3. This sentence previously stated:

“A significant difference was observed in the median OS in the MPR (20 months) and non-MPR groups (16 months) respectively (*p* = 0.0138). In addition, the median DFS showed significant difference and in the MPR (18.5 months) and non-MPR groups (12 months), respectively, (*p *< .001) (Figure 2A).”

The corrected sentence appears below:

“A significant difference was observed in the OS between the MPR and non-MPR groups (*p* = 0.0138). In addition, the DFS showed a significant difference between the MPR and non-MPR groups (*p* < .001) (Figure 2A).”

In the published article, there was an error. Some of the statements in the article about the results in Figure 4B were inappropriate.

A correction has been made to **Results**, *Survival*, Line 4-9, Page 5. This sentence previously stated:

“Prognostic evaluation based on the PD-L1 CPS status revealed median OS durations of 22, 19, and 19 months for patients with PD-L1 CPS < 1, 1≤PD-L1 CPS <20, and PD-L1 CPS≥20, respectively (*p* = .3825). Median DFS durations for the three groups were consistently 16 months (*p* = .0244) (Figure 4B).”

The corrected sentence appears below:

“Prognostic evaluation based on the PD-L1 CPS status revealed that there was no significant difference in the OS among patients with PD-L1 CPS < 1, 1≤PD-L1 CPS <20, and PD-L1 CPS≥20 (*p* = .3825). But there was a significant difference in the DFS among the three groups (*p* = .0244) (Figure 4B).”

The authors apologize for this error and state that this does not change the scientific conclusions of the article in any way. The original article has been updated.

