# Supplementary material for: Neoadjuvant tislelizumab combined with chemotherapy in locally advanced oral or oropharyngeal squamous cell carcinoma: a real−world retrospective study
Source: Front Immunol. 2023 Nov 14;14:1282629. doi: 10.3389/fimmu.2023.1282629 (PMC10685444; doi:10.3389/fimmu.2023.1282629)
Supplement: Supplementary file 1 [file DataSheet_1.docx]

Table S1. The relationship between categorical variables and ORR/N-ORR.

|  |  | ORR (N) | N-ORR (N) | OR  (95%CI) | *p* |
| --- | --- | --- | --- | --- | --- |
| Age |  |  |  | 2.222  (0.462-10.682) | 0.529 |
|  | ≤60 | 12 | 8 |  |  |
|  | >60 | 10 | 3 |  |  |
| Sex |  |  |  | 0.421  (0.070-2.550) | 0.632 |
|  | Male | 19 | 8 |  |  |
|  | Female | 3 | 3 |  |  |
| Tumor sites |  |  |  | 6.500  (1.1247-37.484) | 0.064 |
|  | Oral Cavity | 9 | 9 |  |  |
|  | Oropharynx | 13 | 2 |  |  |
| AJCC stage (the eighth edition) |  |  |  | 0.593  (0.098-3.573) | 0.886 |
|  | III | 6 | 2 |  |  |
|  | IV | 16 | 9 |  |  |
| Smoking |  |  |  | 2.222  (0.489-10.089) | 0.514 |
|  | No | 6 | 5 |  |  |
|  | Yes | 16 | 6 |  |  |
| Drinking |  |  |  | 1.458  (0.335-6.347) | 0.900 |
|  | No | 8 | 5 |  |  |
|  | Yes | 14 | 6 |  |  |
| p16 status |  |  |  | NA | 0.534 |
|  | Positive | 3 | 0 |  |  |
|  | Negative | 19 | 11 |  |  |
| PD-L1 TPS |  |  |  | 1.275  (0.242-6.704) | 1.000 |
|  | <1% | 5 | 3 |  |  |
|  | ≥1% | 17 | 8 |  |  |
| Pathological response |  |  |  | 0.175  (0.035-0.868) | 0.064 |
|  | MPR | 15 | 3 |  |  |
|  | N-MPR | 7 | 8 |  |  |
| PD-L1 CPS |  |  |  | NA | 0.676 |
|  | <1 | 2 | 2 |  |  |
|  | 20＞CPS≥1 | 13 | 6 |  |  |
|  | ≥20 | 6 | 4 |  |  |
