# Supplementary material for: Neoadjuvant tislelizumab combined with chemotherapy in locally advanced oral or oropharyngeal squamous cell carcinoma: a real−world retrospective study
Source: Front Immunol. 2023 Nov 14;14:1282629. doi: 10.3389/fimmu.2023.1282629 (PMC10685444; doi:10.3389/fimmu.2023.1282629)
Supplement: Supplementary file 2 [file DataSheet_2.docx]

Table S2. The relationship between categorical variables and MPR/N-MPR.

|  |  | MPR (N) | N-MPR (N) | OR  (95%CI) | *p* |
| --- | --- | --- | --- | --- | --- |
| Age |  |  |  | 2.5  (0.604-10.334) | 0.202 |
|  | ≤60 | 8 | 10 |  |  |
|  | >60 | 10 | 5 |  |  |
| Sex |  |  |  | 0.344  (0.053-2.215) | 0.484 |
|  | Male | 16 | 11 |  |  |
|  | Female | 2 | 4 |  |  |
| Tumor sites |  |  |  | 1.5  (0.375-5.998) | 0.566 |
|  | Oral Cavity | 9 | 9 |  |  |
|  | Oropharynx | 9 | 6 |  |  |
| AJCC stage (the eighth edition) |  |  |  | 0.875  (0.162-4.713) | 1.000 |
|  | III | 4 | 3 |  |  |
|  | IV | 14 | 12 |  |  |
| Smoking |  |  |  | 1 (0.234-4.278) | 1.000 |
|  | No | 6 | 5 | 6 |  |
|  | Yes | 12 | 10 | 5 |  |
| Drinking |  |  |  | 0.625  (0.151-2.586) | 0.515 |
|  | No | 8 | 5 |  |  |
|  | Yes | 10 | 10 |  |  |
| p16 status |  |  |  | 0.571  (0.047-6.999) | 1.000 |
|  | Positive | 2 | 1 |  |  |
|  | Negative | 16 | 14 |  |  |
| PD-L1 TPS |  |  |  | 5.333  (0.885-32.156) | 0.128 |
|  | <1% | 2 | 6 |  |  |
|  | ≥1% | 16 | 9 |  |  |
| Outcome |  |  |  | 0.175  (0.035-0.868) | 0.064 |
|  | ORR | 15 | 7 |  |  |
|  | N-ORR | 3 | 8 |  |  |
| PD-L1 CPS |  |  |  | NA | 0.023 |
|  | <1 | 0 | 4 |  |  |
|  | 20＞CPS≥1 | 10 | 9 |  |  |
|  | ≥20 | 8 | 2 |  |  |
